# Using genome-wide associations to identify metabolic pathways involved in maize aflatoxin accumulation resistance

**DOI:** 10.1186/s12864-015-1874-9

**Published:** 2015-09-03

**Authors:** Juliet D. Tang, Andy Perkins, W. Paul Williams, Marilyn L. Warburton

**Affiliations:** USDA FS Forest Products Laboratory, Durability and Wood Protection, Starkville, MS 39759 USA; Computer Science and Engineering, Mississippi State, MS 39762 USA; USDA ARS Corn Host Plant Resistance Research Unit, Mississippi State, MS 39762 USA

**Keywords:** Host plant resistance, Pathway analysis, Genome-wide association analysis, Aflatoxin, Corn

## Abstract

**Background:**

Aflatoxin is a potent carcinogen that can contaminate grain infected with the fungus *Aspergillus flavus.* However, resistance to aflatoxin accumulation in maize is a complex trait with low heritability. Here, two complementary analyses were performed to better understand the mechanisms involved. The first coupled results of a genome-wide association study (GWAS) that accounted for linkage disequilibrium among single nucleotide polymorphisms (SNPs) with gene-set enrichment for a pathway-based approach. The rationale was that the cumulative effects of genes in a pathway would give insight into genetic differences that distinguish resistant from susceptible lines of maize. The second involved finding non-pathway genes close to the most significant SNP-trait associations with the greatest effect on reducing aflatoxin in multiple environments. Unlike conventional GWAS, the latter analysis emphasized multiple aspects of SNP-trait associations rather than just significance and was performed because of the high genotype x environment variability exhibited by this trait.

**Results:**

The most significant metabolic pathway identified was jasmonic acid (JA) biosynthesis. Specifically, there was at least one allelic variant for each step in the JA biosynthesis pathway that conferred an incremental decrease to the level of aflatoxin observed among the inbred lines in the GWAS panel. Several non-pathway genes were also consistently associated with lowered aflatoxin levels. Those with predicted functions related to defense were: leucine-rich repeat protein kinase, expansin B3, reversion-to-ethylene sensitivity1, adaptor protein complex2, and a multidrug and toxic compound extrusion protein.

**Conclusions:**

Our genetic analysis provided strong evidence for several genes that were associated with aflatoxin resistance. Inbred lines that exhibited lower levels of aflatoxin accumulation tended to share similar haplotypes for genes specifically in the pathway of JA biosynthesis, along with several non-pathway genes with putative defense-related functions. Knowledge gained from these two complementary analyses has improved our understanding of population differences in aflatoxin resistance.

**Electronic supplementary material:**

The online version of this article (doi:10.1186/s12864-015-1874-9) contains supplementary material, which is available to authorized users.

## Background

*Aspergillus flavus* is one of the causative agents of ear rot in maize. Although infection does not typically reduce yield in temperate environments, the grain can become contaminated with aflatoxin, a polyketide secondary metabolite produced by the fungus that is highly toxic to humans and animals even in minute amounts [1]. In the USA, action levels are 20–300 ppb for animal feed [2] and 0.5 ppb for aflatoxin in fluid milk products [3]. Thus, aflatoxin contamination of maize poses a serious health and economic burden worldwide**.** One promising solution that would mitigate the damage is breeding for host plant resistance.

Most of the known resistance to aflatoxin accumulation in maize has been found in tropical lines, typically with Tuxpan or Tuxpeño in their pedigree [4]. Analysis of bi-parental mapping populations derived from a few of these tropical lines, including CML322 [5], Mp313E [6, 7], and Mp715 [8], has identified many quantitative trait loci (QTL) on all chromosomes but 9 and 10. Narrowing the QTL to single loci with major effects, however, has proved difficult. The QTL encompass large regions, exhibit low to moderate heritability, and are characterized by high genotype x environment interactions [4, 9, 10]. Therefore, host plant improvement by introgression of resistance into temperate lines adapted to the major corn production areas in the US, China, and Europe, has not been efficient.

A complementary approach to QTL mapping is association mapping, which relies on historical recombination events of many different lineages for the discovery of markers linked to the trait of interest. In a genome-wide association study (GWAS) of maize aflatoxin resistance, an association mapping panel of 287 inbred lines was genotyped by sequencing (GBS) and phenotyped for aflatoxin content in testcrossed replicated field experiments [4]. Whole genome association analysis of the data yielded eight single nucleotide polymorphism (SNP)-trait associations that were better than the threshold set for the false discovery rate (FDR) [11]**.** These eight SNPs fell within the sequence of two genes that had conserved domains for DNA methyltransferase and C2H2-like zinc finger protein, and a third gene which was an expressed protein of unknown function. Many more will have been missed, however, as many genes may be expressed only in specific genetic backgrounds, possibly because they are part of a pathway and rely on specific haplotypes at other loci. Thus, the positive alleles of these resistance genes may only be useful when found in combination with the positive alleles of other genes in the same pathway.

In addition to missing true positives due to genetic background or environmental variation, the statistical power of GWAS is limited by strict levels set for FDR and by insufficient numbers of high-frequency polymorphisms found in most panels. FDR helps compensate for multiple testing effects, since a single trait is tested for association against very large numbers of polymorphisms. The candidate gene method of association analysis aims to improve the odds of identifying the most important alleles by genotyping or resequencing only those genes considered to have a high probability of association with the phenotype of interest within the germplasm being tested [12]. This may be done to validate GWAS results, or to find associations that GWAS missed. Many successful studies of candidate gene association analysis in maize have been published to improve traits like flowering time [13] and kernel traits like starch production [14], β-carotene content [15], and provitamin A biofortification [16].

Metabolic pathway analysis focuses on the combined effects of many genes that are grouped according to their shared biological function. This is another promising approach that can complement the most significant SNP-trait associations or give clues to the genetic basis of a trait [17]. Although originally developed to study differences in gene expression data for medically important diseases [18], pathway analysis has been used with association mapping to find biological insights missed when focusing on only one or a few genes that have the highest associations with the trait of interest. In addition, biologically relevant pathways can be used to determine candidate genes for association analysis or to interpret large data sets produced by other high-throughput approaches like RNA sequencing, proteomics, and metabolomics.

Pathway-based approaches are now used routinely to study human disease [19–21], but published reports on pathway analysis of GWAS data in plants are still non-existent. Combining the aflatoxin GWAS data in a pathway analysis jointly considers all genetic sequences positively associated with *A. flavus* infection and aflatoxin accumulation resistance; thus, pathways may be highlighted which lead to mechanisms for fungal resistance and those that discourage fungi in the maize kernel from producing deleterious aflatoxin. Identification of these genes will eventually lead to more efficient breeding procedures and development of maize hybrids with resistance to aflatoxin accumulation. A better understanding of pathways involved in resistance will also advance our broader understanding of plant defense mechanisms against other opportunistic saprobic fungi. Therefore, the primary objective of this study was to identify metabolic pathways and pathway genes underlying aflatoxin resistance by accounting for linkage disequilibrium among SNPs from a large-scale GWAS study. A second separate, but complementary objective was to identify genes within 1 Kb of significant SNP-trait associations whose effects for lowering aflatoxin recurred in multiple environments. Unlike conventional GWAS, this analysis emphasized multiple aspects of SNP-trait associations rather than just significance and was performed because of the high genotype x environment variability exhibited by this trait. By synthesizing the combined results, we hope to better understand the relationships that connect metabolic pathway and non-pathway genes in maize aflatoxin resistance. The joint analysis of all genes in this manner is expected to uncover new mechanisms that improve resistance to aflatoxin accumulation in maize.

## Results and discussion

### GWAS

Among the 287 inbred maize lines, TASSEL calculated SNP-trait associations for 261,183 SNPs [11]. Of these, 45.8 % of the SNP allele calls were imputed from the regional haplotype. The range of association *p,* effect, and R^2^ values were 2.87E^−10^ to 1.0, −2.55 to 3.46, and 6.4E^−14^ to 0.3, respectively. Sorting output of the linkage disequilibrium values between pairs of SNPs in ascending order by the position of SNP 1 or 2 produced linkages from the reference SNP in the upstream and downstream directions, respectively. Due to size limitations, the TASSEL output files could not be included in the supplement but they are available upon request.

### SNP to gene algorithm for the pathway analysis

A plot of pairwise linkage disequilibrium values—log(*p*) against R^2^ showed that the most significant linkages between a reference SNP and its linked SNP occurred for R^2^ > 0.8 (Additional file 1: Figure S1). Based on this distribution, 0.8 was chosen as the threshold to define linkage. The frequency of linkage types were: 46.3 % unlinked (type 0), 33.5 % reference SNP linked to single SNP (type 1), 19.1 % reference SNP linked to a SNP block where the associations in the block had a majority effect sign, that is the majority of associations had either a positive or negative effect (type ≥ 2, the type number here refers to the number of linked SNPs in the block), and 1.1 % where the block had no majority effect sign (equal numbers of associations with positive and negative effects, type −1) (Additional file 2: Figure S2). A comparison of the effect signs in linkage groups (reference SNP linked to a single SNP or SNP block), showed that 84 % of the linkage types shared the same sign (between reference SNP and linked SNP or between reference SNP and majority effect sign for the SNP block), while 16 % did not. The SNP to gene algorithm was designed to account for all possibilities present.

**Fig. 1:**
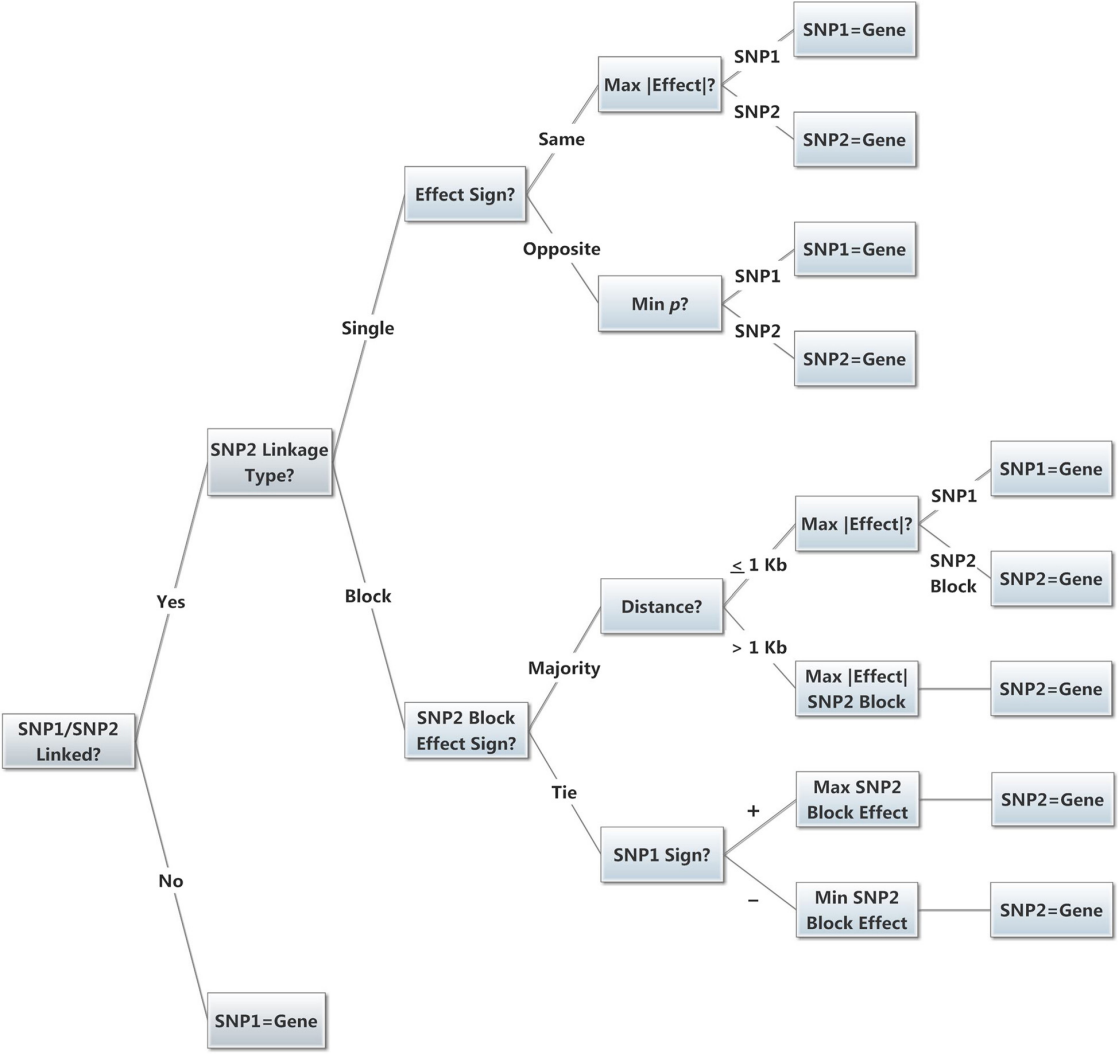
Decision tree to find the tagSNP and gene for the GWAS results based on linkage disequilibrium values. The tagSNP is at the terminal branch of the tree, SNP1 is the reference SNP, SNP2 Block is a block of SNPs linked to SNP1 (R^2^ > 0.8), and SNP2 is a found SNP within the SNP2 Block based on the decisions made by the algorithm. The values of the association effect and significance (*p*) were obtained from the GWAS. Once a tagSNP was identified, it was assumed that the gene(s) causing the association was within 1 Kb

The steps of the decision tree used to find the tagSNP and gene are detailed in Fig. 1. If there was no linkage (type 0), then the reference SNP (labeled SNP1 in Fig. 1) was the tagSNP used to find the gene. For linkage type 1 (path labeled Single in Fig. 1), if SNP1 and the linked SNP (labeled SNP2 in Fig. 1) had the same effect sign, the SNP with the maximum absolute value of the effect value (|effect|) was designated as the tagSNP. If the SNP1 and SNP2 had opposite effect signs, the SNP with the most significant association was assigned the tagSNP. For linkage types ≥ 2 (path labeled Block in Fig. 1), two branches were possible depending upon whether the SNP2 block had a majority effect sign or not. If yes, then the distance between SNP2 and the SNP2 block was examined. If the distance was ≤ 1 Kb then the tagSNP was the SNP with the maximum |effect| among SNP1 and SNPs in the SNP2 block. If the distance was > 1 Kb, then the tagSNP was the SNP with the maximum |effect| from the SNPs in the SNP2 block only. If the number of SNPs with positive and negative effect signs in the SNP2 block was tied, then the effect sign of SNP1 was used to break the tie and find the SNP in the SNP2 block with the maximum (labeled + in Fig. 1) or minimum (labeled—in Fig. 1) effect value. Once the tagSNP was identified, the associated gene(s) was assumed to be within 1 Kb. This search distance was based on our finding that the majority (62 %) of linkages between two SNPs (linkage disquilibrium R^2^ > 0.8) was within 1 Kb (Additional file 3: Figure S3). Rapid decay of average linkage disequilibrium is typical for maize, especially for tropical germplasm, and has been studied in detail by Romay et al. [22]. The association *p*, effect, and R^2^ values of the tagSNP were then assigned to the located gene. A total of 25,246 tagSNPs were used to locate 25,404 genes.

### Validity of the GWAS to pathway pipeline

Kernel color, which is a trait known to involve the pathway PWY-6475-1 (*trans*-lycopene biosynthesis II), was used to test the performance of the GWAS to pathway pipeline. All steps were similar to those used for the pipeline for aflatoxin resistance except (1) the trait was fraction of yellow kernels, where 1 was all yellow and 0 was all white, and (2) the gene effect scores were ranked from yellow to white for the enrichment score calculation. The top two pathways found in this verification analysis were PWY-6299 (aldehyde oxidation I, *p* = 0.006) and the expected pathway, PWY-6475-1 (*trans*-lycopene biosynthesis II, *p* = 0.009). PWY-6299 was a one step oxidation of the precursors (abscisic aldehyde, aldehyde, or benzaldehyde) to the corresponding acid that had 8 genes contributing to the enrichment score. The involvement of this pathway in kernel color is unknown, but the yellow pigment of corn kernels are carotenoids and abscisic acid is biosynthesized from C_40_ carotenoid precursors. The expected pathway, PWY-6475-1, has 9 sequential reactions that begin with two molecules of geranylgeranyl diphosphate, which are condensed by phytoene synthase to phytoene, the first committed step of carotenoid biosynthesis. Based on the ranks of the effect values, there were three of seven genes that contributed the most to the enrichment score. The genes and their MaizeCyc enzyme annotations were : GRMZM2G300348 (PSY1, phytoene synthase), GRMZM2G108457 (carotenoid isomerase 1), and GRMZM2G454952 (ZDS, zeta-carotene desaturase). These three genes were unique to PWY-6475-1, that is their reactions were not mapped to any other MaizeCyc pathway. Further, PSY1, which confers yellow color to endosperm, has been shown to be essential for carotenoid biosynthesis [23]. Therefore, the test results appeared to support the validity of our GWAS to pathways analysis.

### Most significant pathways

**Fig. 2:**
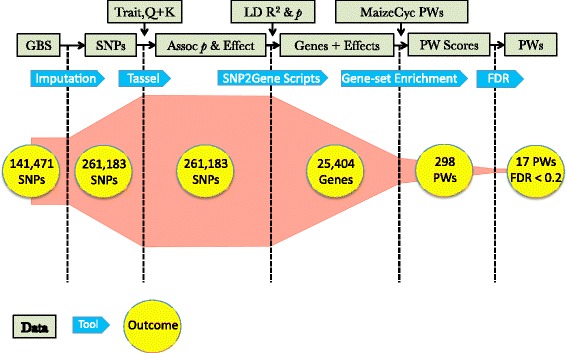
Analysis pipeline that coupled the GWAS, linkage disequilibrium, and pathway analyses. The outcome or size of the data set following each step is indicated. Assoc, association; LD, linkage disequilibrium; Q + K, genetic marker-based kinship matrix plus population structure files for the mixed linear model analysis implemented by TASSEL; PW, pathways

**Fig. 3:**
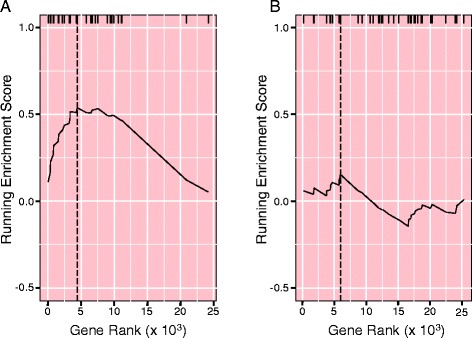
Graphs of the running enrichment score calculation for (**a**) PWY-735 (JA biosynthesis pathway) and (**b**) ETHYL-PWY (ethylene biosynthesis pathway). Genes were ranked in ascending order by their effect scores. Hash marks at the top of the graph denote the ranks of genes in the pathway. The pathway enrichment score coincided with the maximum running enrichment score and is marked by the dashed vertical line

Figure 2 summarizes the steps in the GWAS to pathways pipeline for grain aflatoxin levels and includes data inputs for each tool and their outcomes. Of the 25,404 gene associations found, 2880 of the genes mapped to the 298 MaizeCyc pathways that had five or more genes. Of these, 17 pathways (containing 243 genes) had enrichment scores better than FDR < 0.2 (Table 1). Graphs of two pathways for the biosynthesis of plant hormones illustrate how the values of the running enrichment score changed with gene rank (Fig. 3). The jasmonic acid biosynthesis pathway (PWY-735, Fig. 3a) had a high enrichment score (0.54) because the genes in the pathway (denoted by the hash marks along the top of the graph) were among the topmost ranks and thus increased the value of the enrichment score. This contrasted with the ethylene biosynthesis pathway (ETHYL-PWY, Fig. 3b), which had a lower enrichment score (0.15) and fewer genes in the topmost ranks. After normalization of the enrichment score, PWY-735 and ETHYL-PWY were ranked number 1 and 153 out of 298, respectively.

**Table 1:** Summary of the gene-set enrichment analysis for pathways with FDR < 0.2

MaizeCyc ID	PW Name	NES	*p*	*q*	Genes^a^
PWY-735	jasmonic acid biosynthesis	4.55	2.63E-6	0.001	28
PWY-6124	inosine-5′-phosphate biosynthesis II	3.18	0.0007	0.109	8
PWY-5136	fatty acid β-oxidation II (core pathway)	2.85	0.0022	0.163	25
ARGASEDEG-PWY	arginine degradation I (arginase pathway)	2.79	0.0026	0.163	14
FOLSYN-PWY-1	superpathway of tetrahydrofolate biosynthesis	2.68	0.0037	0.163	15
PWY-3001	isoleucine biosynthesis I	2.64	0.0041	0.163	65
SULFATE-CYS-PWY	superpathway of sulfate assimilation and cysteine biosynthesis	2.61	0.0046	0.163	24
BRANCHED-CHAIN-AA-SYN-PWY	superpathway of leucine, valine, and isoleucine biosynthesis	2.55	0.0054	0.163	38
PWY-5409	divinyl ether biosynthesis II	2.53	0.0056	0.163	7
PWY-6435	4-hydroxybenzoate biosynthesis V	2.53	0.0057	0.163	20
PWY-3461	tyrosine biosynthesis II	2.50	0.0063	0.163	7
PWY-561	superpathway of glyoxylate cycle	2.46	0.0069	0.163	59
PWY-6040	chlorogenic acid biosynthesis II	2.43	0.0076	0.163	5
PWY-4221-1	superpathway of pantothenate and coenzyme A biosynthesis II	2.43	0.0076	0.163	43
THRESYN-PWY	threonine biosynthesis	2.40	0.0083	0.165	41
LEUSYN-PWY	leucine biosynthesis	2.37	0.0090	0.167	21
PWY-5481	pyruvate fermentation to lactate	2.33	0.0098	0.172	8

*ID* identifier, *PW* pathway, *NES* normalized enrichment score

^a^The number of genes that were mapped to a pathway and contributed to the enrichment score calculation

The normalized enrichment score, *p*, *q*, and gene count for the 17 top ranking pathways (FDR < 0.2) are listed in Table 1. A summary of the gene identifiers belonging to these pathways with their tagSNPs, association statistics, and alleles are provided (Additional file 4: Table S1). The jasmonic acid (JA) biosynthesis pathway (PWY-735) was by far the most significant (*p* = 2.63E-6, FDR = 0.001). JA is a cyclopentanone oxylipin biosynthesized through the allene oxide synthase (AOS) branch of the lipoxygenase (LOX) pathway [24]. Our results indicate that the allelic variation found among the genes involved in the biosynthesis of JA were critical for determining the level of resistance to aflatoxin contamination in kernels.

JA signaling has known roles for increasing resistance to necrotrophic pathogens [25]. Plant-derived oxylipins like 13*S-*hydroperoxyoctadecadienoic acid (13*S*-HPODE) and 9*S-*HPODE are known to mimic fungal oxylipins [26] and induce increased conidiation and increased production of aflatoxin when applied to cultures of *Aspergillus* species [27, 28]. Although our analysis did not examine how the allelic variation affected kernel levels of JA, fatty acid precursors, or other 9- and 13-LOX derivatives, it is conceivable that resistance was correlated with changes in flux to the various branches of the LOX pathway that favored JA biosynthesis over other oxylipins.

Increases in foliar levels of JA have also been associated with defense against herbivores. In a comparison of herbivore-resistant (Mp708) and susceptible (Tx601) lines of maize, higher foliar levels of JA and the cyclopentenone intermediate, 12-oxophytodienoate, were found in the resistant line with levels increasing after exposure to fall armyworm larvae [29]. Maize is similar to other plants in that exogenous application of JA to leaves induced the accumulation of defense-related compounds like phytoalexins, mimicking the accumulation observed after fungal inoculation or wounding [30]. In addition, the timing of the increase in endogenous JA levels after damage and fungal inoculation have supported a role for the JA signaling pathway in initiating localized plant defense mechanisms [30].

PWY-735 had 28 genes contributing to the calculation of the enrichment score in 11 reaction types. The first reaction (EC 1.13.11.12) is the oxidation of the fatty acid, α-linolenate to form 13*S*-HPODE by LOX. Step 2 (EC 4.2.1.92) forms the epoxide, 12,13-epoxylinolenate. This reaction is unique to PWY-735 and can be catalyzed by hydroperoxide dehydratase (HD), AOS, and cytochrome P450 (CYP450). The epoxide, being unstable, undergoes cyclization by allene oxide cyclase (AOC, EC 5.3.99.6) to produce the cyclopentenone intermediate 12-oxophytodienoate (step 3). Reduction in step 4 by 12-oxophytodienoate reductase (OPR, EC 1.3.1.42) is followed by addition of Coenzyme A (step 5) and three rounds of β-oxidation (steps 6–9, repeated three times) to produce jasmonyl-CoA. Step 6 is a dehydrogenation reaction (EC 1.3.3.6) catalyzed by acyl-CoA oxidase (ACO), acyl-CoA dehydrogenase (ACAD), and dodecenyl-CoA isomerase (DCAI) that produces the corresponding *trans* enoyl-CoA. In step 7 (EC 4.2.1.17: enoyl-CoA hydratase, ECH; enoyl-CoA hydratase2, ECH2; and 3-hydroxybutyryl-CoA dehydrogenase, BUDH), water is added to the enoyl group, and in step 8 (EC 1.1.1.35: enoyl-CoA hydratase2, ECH2; and 3-hydroxyacyl-CoA-dehydrogenase, HAD), dehydrogenation converts the hydroxyacyl-CoA to the keto-acyl-CoA. Thiolytic cleavage in step 9 (EC 2.3.1.16: acetyl-CoA C-acyltransferase, ACAA) forms an acyl-CoA that is two carbons shorter. Hydrolysis removes the Coenzyme A moiety (step 10), and a configuration change at one of the two stereocenters (step 11) ends the pathway with the formation of the prohormone (−)-jasmonate. The reactions lacking evidence in maize are catalyzed by EC 6.2.1 in step 5 (acid thiol ligase), EC 3.1.2.20 in step 10 (acyl-CoA hydrolase), and the conformation change in step 11.

### Most significant pathway genes

**Fig. 4:**
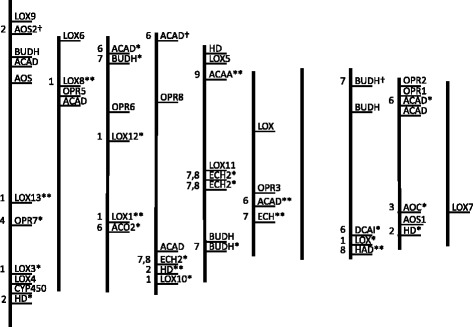
Relative positions of the genes in PWY-735 on the maize chromosomes. Thick vertical lines depict chromosomes 1–10 (left to right). Genes with notations were associated with a tagSNP and contributed to the enrichment score calculation. Notations: *gene had a negative effect value, **gene had *p* < 0.1 and effect < −0.1, † gene had a positive effect value. Numbers to the left of the chromosome refer to the pathway step catalyzed. Genes that lacked notations did not contribute to the enrichment score because they lacked polymorphisms among the lines in the association panel or lacked sequenced reads from the region. See text for abbreviations of gene function and EC number of the reaction catalyzed

For each of the nine reaction types of PWY-735 with evidence in maize, there was at least one gene that had an associated tagSNP with a negative effect value for decreasing aflatoxin contamination (Fig. 4). Thus, there was at least one allelic variant for each of these nine steps in the JA biosynthesis pathway that conferred an incremental decrease to levels of aflatoxin observed among the association panel. Genes that contributed the most to the enrichment score (*p* < 0.1 and effect < −0.1 and marked with a double asterisk in Fig. 4) appear in Table 2. They were mapped to steps 1, 2, and 6–9 (fatty acid β-oxidation). Genes for all three LOXs (LOX1, LOX8, and LOX13), HD (unique to PWY-735), and ACAA all fell within previously described QTL for resistance to aflatoxin contamination in maize [5, 31]. Interestingly, the gene for HD fell within a cluster with ECH2 and LOX10 on chromosome 4 (Fig. 4). A suspected gene cluster in this region was reported by Mideros et al. [5], whose meta-analysis of several previous QTL mapping studies found 12 independent QTLs, 7 in bins 4.07–4.08 and 5 in bin 4.09, with the largest-effect QTL in bin 4.08. ACAA was common to three other pathways (PYW-5136, fatty acid β-oxidation II; PWY-561, superpathway of glyoxylate cycle; and PWY-6435, 4-hydroxybenzoate biosynthesis V).

**Table 2:** Annotation and QTL evidence for genes from the JA biosynthesis pathway with the most significant associations and greatest effects^a^ on lowering aflatoxin accumulation

tagSNP	*p*	R^b^	Effect	Gene Identifier	MaizeCyc Annotation	Step^c^	Bin	QTL^d^
S1_188114572	0.059	0.01	–0.23	GRMZM5G822593	lipoxygenase13	1	1.06	mqtl1_5 mqtl1_6
S2_45193435	0.047	0.01	–0.24	GRMZM2G104843	lipoxygenase8	1	2.04	mqtl2_2
S3_168834972	0.076	0.03	–0.36	GRMZM2G156861	lipoxygenase1	1	3.05	mqtl3_3
S4_230398767	0.007	0.02	–0.37	GRMZM2G168404	hydroperoxide dehydratase	2	4.09	mqtl4_8
S5_31133128	0.002	0.04	–0.60	GRMZM2G110201	acetyl-CoA C-acyltransferase	9	5.03	Mp715
S6_124147817	0.065	0.01	–0.15	GRMZM2G002959	acyl-CoA dehydrogenase	6	6.05	None
S6_138501372	0.062	0.02	–0.20	GRMZM2G117357	enoyl-CoA hydratase	7	6.05	None
S8_171705472	0.047	0.03	–0.27	GRMZM2G106250	3-hydroxyacyl-CoA dehydrogenase	8	8.08	None

^a^Subset had association *p* < 0.1 and effect < −0.1

^b^Proportion of the phenotypic variation accounted for by the tagSNP

^c^ Pathway step described in the text

^d^Data for QTL (resistant parent listed) and meta-QTL (mqtl) for resistance to *Aspergillus* species were compiled from Mideros et al. Supplementary Table 8 [5] and Warburton and Williams [31]. TagSNP was in or within 2 Mb of QTL

Functional analysis of heterologously expressed LOX1 from maize showed that it is a non-traditional LOX exhibiting both 9- and 13-LOX activities [32]. In maize seedlings, *LOX1* gene expression is induced by wounding and exogenous application of methyl jasmonate [32]. When gene expression levels of *LOX1, LOX3,* and *AOS1* were quantified in Mp708 compared to Tx601, all three exhibited constitutively higher expression levels in the resistant line compared to the susceptible line before and after 24 h of feeding by fall armyworm, but relative fold changes were about an order of magnitude higher for *LOX1* [29]. Other studies have demonstrated that chloroplast-localized LOX1 [32] and LOX8 [33] are responsible for wound-induced JA production in maize leaves. LOX13 has no known functional role, but shares highest sequence homology with LOX10 [33], a 13-LOX that provides precursors for the production of the green leaf volatiles (hydroperoxide lyase branch of the LOX pathway), as well as semiochemicals that recruit predators and parasites to the wounded area. In addition, LOX10 mediates JA production by LOX8 because LOX8 is dependent upon signaling from LOX10-derived oxylipins [33].

Even though the remaining pathways in Table 1 had FDR values between 0.1 and 0.2, several could be associated with decreasing levels of aflatoxin because of their relationships with JA. Among the biologically active forms of JA, JA-isoleucine is the most potent signaling form [34, 35]. This may explain why we detected PWY-3001 (isoleucine biosynthesis I), BRANCHED CHAIN-AA-SYN-PWY (superpathway of leucine, valine, and isoleucine biosynthesis), and THRESYN-PWY (threonine biosynthesis pathway). The first two pathways produce isoleucine, and the third produces threonine, a precursor for isoleucine biosynthesis.

Detection of PWY-5409 (biosynthesis of divinyl ethers II) in Table 1 may have occurred because divinyl ethers are oxylipin secondary metabolites, biosynthesized through the divinyl ether synthase branch of the LOX pathway [24]. In potato, divinyl ethers inhibit growth of the fungal pathogen, *Phytophthora infestans,* and accumulate more in resistant than susceptible cultivars [36].

Another compound involved in defense is glutathione, a potent antioxidant that is induced by JA in response to oxidative stress [37]. Glutathione can also influence basal levels of JA gene expression and JA signaling strength [38]. These roles may explain the associations of two pathways that provide precursors for glutathione production, the ARGASEDEG-PWY, which produces glutamate, and the SULFATE-CYS-PWY, a superpathway for sulfate assimilation and cysteine biosynthesis (Table 1).

Other pathways that had possible associations because of their roles in plant defense are PWY-6435 (4-hydroxybenzoate biosynthesis V) and PWY-6040 (chlorogenic acid biosynthesis II) (Table 1). Both are phenylpropanoid derivatives found in plant cell walls. The phenolic acid, 4-hydroxybenzoate, accumulates along with other aromatic compounds after pathogen infection [39, 40]. Levels of chlorogenic acid (5-*O*-caffeoylquinic acid), one of the most important antimicrobials found in cell walls, can be constitutively isolated from resistant cultivars or induced by pathogen infection depending upon the plant species [41, 42].

### Individual genes with significant effects on aflatoxin resistance

After applying the sequential filters, 13 genes were found flanking 25 SNPs that had the greatest associations in multiple environments for lowering aflatoxin levels. All but two had annotations and six were located in previously described QTL (Table 3). The annotations (from *Arabidopsis thaliana* gene orthologs) related to defense were leucine-rich repeat protein kinase (LRRPK), expansin B3 (EXPB3), reversion-to-ethylene sensitivity1 (RTE1), the α-subunit of the adaptor protein complex2 (AP-2), and a multidrug and toxic compound extrusion protein (MATE).

**Table 3:** Annotation and QTL evidence for individual genes with the most consistent associations for lowering aflatoxin resistance^a^

Marker	*p*	R^b^	Effect	Geneid	TAIR Annotation^c^	Bin	QTL^d^
S1_45772203	0.0051	0.07	–0.69	GRMZM2G135359	LRRPK family protein	1.03	mqtl1_3
S2_23556305	0.0009	0.10	–0.77	GRMZM2G078279	expansin B3	2.03	mqtl2_1
S2_37133737	0.0001	0.07	–0.57	GRMZM2G057637	None	2.04	None
S3_23493748	0.0017	0.06	–0.83	GRMZM2G041352	endonuclease 2	3.04	mqtl3_2
S4_238436842	0.0009	0.07	–0.61	GRMZM2G174481	NAD(P)-OR superfamily protein	4.10	mqtl4_9
S5_193538196	0.0004	0.09	–0.76	GRMZM2G165601	None	5.05	None
S5_213916936	0.0061	0.07	–0.75	GRMZM2G469142	AlgP	5.08	mqtl5_6
S6_5158838	0.0023	0.08	–0.54	GRMZM2G125081	LRRPK family protein	6.00	None
S6_5158838	0.0023	0.08	–0.54	GRMZM2G125138	glycine-rich protein	6.00	None
S6_161709273	0.0040	0.07	–0.82	GRMZM2G121208	RTE1	6.06	mqtl6_8
S9_154403351	0.0002	0.08	–0.62	GRMZM2G092741	AP-2, α subunit	9.07	None
S9_154245175	0.0042	0.06	–0.68	GRMZM2G303312	ECT4	9.07	None
S10_2433643	0.0003	0.08	–0.61	GRMZM2G151903	MATE efflux family protein	10.00	None

^a^Two sequential filters applied. Filter 1 = *p* < 0.01, effect < −0.2, and R^2^ > 0.04. Filter 2 = R^2^ > 0.06 and effect < −0.5

^b^Proportion of the phenotypic variation accounted for by the tagSNP

^c^Abbreviations: TAIR, The *Arabidopsis* Information Resource; LRRPK, leucine-rich repeat protein kinase; NAD(P)-OR, NAD(P)-linked oxidoreductase; AlgP, alginate regulatory protein; RTE1, reversion-to-ethylene sensitivity1; AP, adaptor protein complex; ECT4, evolutionarily conserved C-terminal region 4; MATE, multidrug and toxic compound extrusion. The AlgP annotation was from rice

^d^Data for QTL (resistant parent listed) and meta-QTL (mqtl) number for resistance to *Aspergillus* species were compiled from Mideros et al. Supplementary Table 8 [5]and Warburton and Williams [31]. TagSNP was either in or within 2 Mb of QTL

LRRPKs comprise the largest sub-family of receptor-like kinases. Despite their abundance, only a handful have been studied in depth with diverse signaling roles related to development and pathogen recognition in host defense [43]. The expression of a gene for EXPB3 was one of several genes down-regulated by cyclopentenones and involved in cell wall remodeling [44]. Cyclopentenones (e.g. 12-oxophytodienoate and phytoprostanes) like the cyclopentanone JA are potent signaling compounds that accumulate in response to wounding and pathogen infection [45]. RTE1 is a negative regulator of ethylene signaling found to interact with at least one of the ethylene-responsive receptors [46]. AP-2 binds cargo into clathrin-coated pits during endocytosis, an essential process cells use to internalize nutrients, communicate with the exterior, recycle plasma membrane, and mediate plant-microbe interactions [47]. The antiporter activity of MATE family proteins have known roles for moving xenobiotics, cations, organic acids, and secondary metabolites out of the cytoplasm to the exterior or into vacuoles [48].

## Conclusions

Although resistance to aflatoxin accumulation in maize kernels is a quantitative trait with high genotype x environment variability, we were able to apply GWAS data to a pathway-based approach, which groups genes based on their shared biological function, to find genetic differences that distinguish resistant and susceptible lines of maize. Most notably, we determined that the allelic variation found among the genes involved in the biosynthesis of JA were highly associated with the levels of aflatoxin observed among the panel of 287 inbred lines examined. Moreover, we detected at least one allelic variant for each of the nine reaction types in the JA biosynthesis pathway that conferred an incremental decrease to the overall levels of aflatoxin observed. We were also able to identify non-pathway genes with putative defense-related functions in our second approach, which used a conventional GWAS analysis, but emphasized SNP-trait associations that consistently lowered aflatoxin levels in multiple environments. Knowledge gained from these two complementary analyses has improved our understanding of population differences in aflatoxin resistance and, following additional verification, will provide markers for host plant improvement by introgression. To this end, the candidate gene method of association analysis and the construction of near isogenic, transgenic, or mutant plants will be employed to validate the more important alleles identified and how they affect aflatoxin accumulation.

## Methods

### GWAS

An association mapping panel of 287 maize inbred lines with varying levels of resistance to aflatoxin accumulation was assembled and characterized by Warburton et al. [4]. Following GBS [49], GWAS was performed and reported in Warburton et al. [4, 11]. Briefly, testcrosses of the plants were deployed in a randomized complete block design with three replications in seven different environments spread over Texas and Mississippi in 2009 and 2010. Ears were inoculated with *Aspergillus flavus* (strain NRRL 3357) using the side-needle technique [50] seven days after half of the primary ears showed silk. At harvest, the dried and ground grain (50 g samples) was phenotyped for aflatoxin content (measured in ng/g) using the Vicam AflaTest (VICAM, Watertown, MA). The least squared means of the natural log transformed values of aflatoxin from each environment and the average over environments were calculated. Analysis of the SNP-trait associations in terms of their significance (*p*)*,* correlation (R^2^ or proportion of the phenotypic variation accounted for), and effect values were performed with a mixed linear model approach implemented by TASSEL [51] with a minor allele frequency setting > 0.05. Effect values could be positive or negative and indicated the relative amount that a SNP increased or decreased aflatoxin levels, respectively. TASSEL also calculated linkage disequilibrium (*D’,* R^2^, and *p*) [52] between each marker SNP (denoted as the reference SNP) and its closest neighboring SNPs (50 upstream and 50 downstream). Since both SNP-trait associations and linkage disequilibrium have R^2^ and *p* values that were used at different times in the analyses, this text will state the context to avoid confusion.

### SNP to gene algorithm for the pathway analysis

The goal of the SNP to gene algorithm was to identify the tagSNP from SNP linkage groups and then determine if there was one (or more) nearby gene(s), presumably causing the association. The tagSNP served two main purposes: 1) by accounting for linkage, it reduced the dimensionality of the dataset, and 2) it assigned the SNP-trait association with the largest |effect| to the gene along with other attributes including R^2^ and *p*.

The threshold for linkage was determined from a plot of linkage disequilibrium values, —log(*p*) against R^2^. The decisions implemented by the algorithm were based on: 1) linkage type (no linkage versus linked to one or more SNPs in a block), 2) the majority sign (positive or negative) of the SNP-trait association effects in the linkage block versus the effect and association *p* values for the reference SNP, and 3) distance between linked SNPs. Once a tagSNP was identified, the search window for the causative gene was set to ± 1 Kb, which was based on a histogram of distances between linked SNPs. The association effect and *p* values of the tagSNP were then assigned to the identified gene. The linkage threshold determination and gene search window were taken from plots made for chromosome 1, which appeared to be typical for all 10 chromosomes.

### Pathways

For the pathway analysis, the SNP to gene algorithm was run with associations for the phenotype from the average environment only. Gene-set enrichment calculations were performed according to Subramanian et al. [18]. Genes were grouped into pathways as outlined by MaizeCyc v2.1 [53]. Only pathways with five or more genes (298 pathways total) were considered to reduce bias from small sample size. A pathway in MaizeCyc could also be a superpathway, which is composed of multiple individual pathways and may have reactions of its own. Genes were ranked by their effects (negative to positive), and a running sum statistic was calculated that increased or decreased if genes were or were not, respectively, in the pathway. The amount of increase was the fraction of genes in the pathway weighted by the |effect|, while the amount of decrease was the fraction of genes not in the pathway. This procedure is similar to calculating a weighted Kolmogorov-Smirnov statistic. The enrichment score (ES) for the pathway was the maximum deviation from zero. The significance of a pathway was determined by taking 1000 permutations of the gene effect values to generate a null distribution for the ES. The null distribution mean (*μ),* and standard deviation (*σ*) served to normalize the ES for the pathway, (*X-μ)/σ*, and pathway *p* values were computed using the pnorm function in R [54]. The values of *p* were then corrected for FDR as calculated by the QVALUE package in R [55]. Genes that contributed the most to the enrichment scores of pathways with FDR < 0.2 were filtered based on thresholds set for gene association and effect values.

### Identifying individual genes with significant effects on aflatoxin resistance

A second separate, but complementary objective was to identify non-pathway genes that were (1) within 1 Kb of SNP-trait associations whose significance exceeded a minimum threshold (*p*), (2) acted consistently to lower aflatoxin levels (negative effect value), where effects recurred in multiple environments, and (3) exceeded a minimum threshold set for the fraction of the phenotypic variation accounted for (R^2^). To implement these criteria, SNP-trait associations from all seven environments plus the average (eight total) were subjected to two sequential filtering steps. The first filter retained SNPs if *p* < 0.01, effect < −0.2, and R^2^ > 0.04, with the three criteria being met in a minimum of three environments; the second filter further reduced the list by keeping only those associations with R^2^ > 0.06 and effect < −0.5. The causative gene was then assumed to be within 1 Kb of the SNP. When there were several SNPs close to the same gene, the SNP with the lowest *p* was assigned to the gene for this analysis. Information regarding tagSNPs, linkage disequilibrium, and pathways were not considered in this analysis.

### Data sources and analysis tools

The *Zea mays* reference sequence (B73 RefGen v2 assembly), canonical gene coordinates (ZmB73_5b_FGS_info.txt), and gene functional annotations (ZmB73_5a_gene_descriptors.txt) were obtained from MaizeSequence.org. The SNP marker data (AllZeaGBSv27_imputed posted on December 18, 2013) [22] was downloaded from the Panzea website (www.panzea.org/lit/data_sets.html). Orthologous gene information for rice and *A. thaliana* (Zmays_181_annotation_info.txt) was retrieved from phytozome.org. The MaizeCyc v2.1 pathway genome database [53] was accessed from www.maizegdb.org. Unless otherwise specified, scripts to perform analyses were written in Perl 5 v16 (www.perl.org). Graphing and statistical analyses were done in R v3.0.2 [54].

## Additional files

Additional file 1: Figure S1. A plot of the chromosome 1 linkage disequilibrium values, −log(*p*) against R^2^, showed that the most significant R^2^ values occurred for R^2^ > 0.8. Based on this observation, 0.8 was chosen as the threshold to define SNP linkage. Points were binned and the number of counts in a bin was denoted by the blue shading. (PDF 14 kb) Additional file 2: Figure S2. Distribution of linkage types for chromosome 1. The frequency of linkage types were: 46.3 % unlinked (type 0), 33.5 % linked to a single SNP (type 1), 19.1 % linked to a block where the block showed a majority effect sign (type ≥ 2, where the type number refers to the number of SNPs in the block), and 1.1 % where the block had no majority effect sign (type −1). (PDF 24 kb) Additional file 3: Figure S3. Histogram of the distance between two linked SNPs for (A) all of chromosome 1, and (B) for the region in (A) where the SNPs were separated by less than 20 Kb. (PDF 41 kb) Additional file 4: Table S1. Association effect and p values for tagSNPs and genes in the 17 pathways with FDR < 0.2. (XLSX 92 kb)
