# Supplementary figures and images for: Using genome-wide associations to identify metabolic pathways involved in maize aflatoxin accumulation resistance
Source: BMC Genomics. 2015 Sep 3;16(1):673. doi: 10.1186/s12864-015-1874-9 (PMC4558830; doi:10.1186/s12864-015-1874-9)

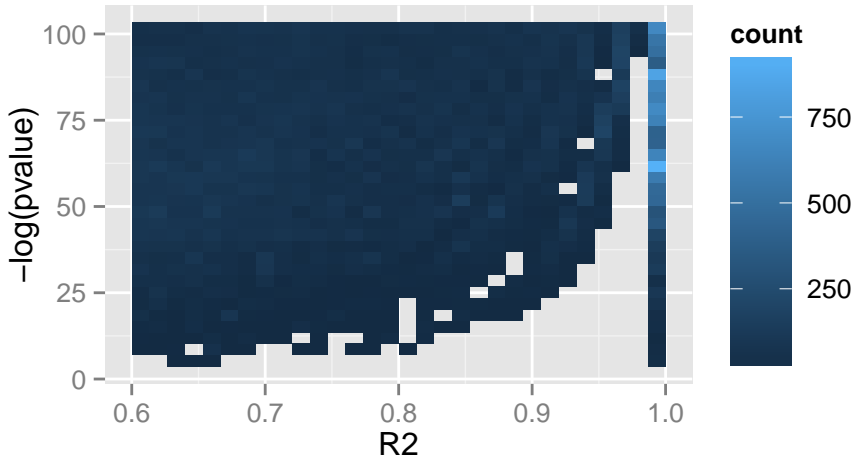

Supplement: Additional file 1: Figure S1. — A plot of the chromosome 1 linkage disequilibrium values, −log(p) against R2, showed that the most significant R2 values occurred for R2 > 0.8. Based on this observation, 0.8 was chosen as the threshold to define SNP linkage. Points were binned and the number of counts in a bin was denoted by the blue shading. (PDF 14 kb) [file 12864_2015_1874_MOESM1_ESM.pdf]

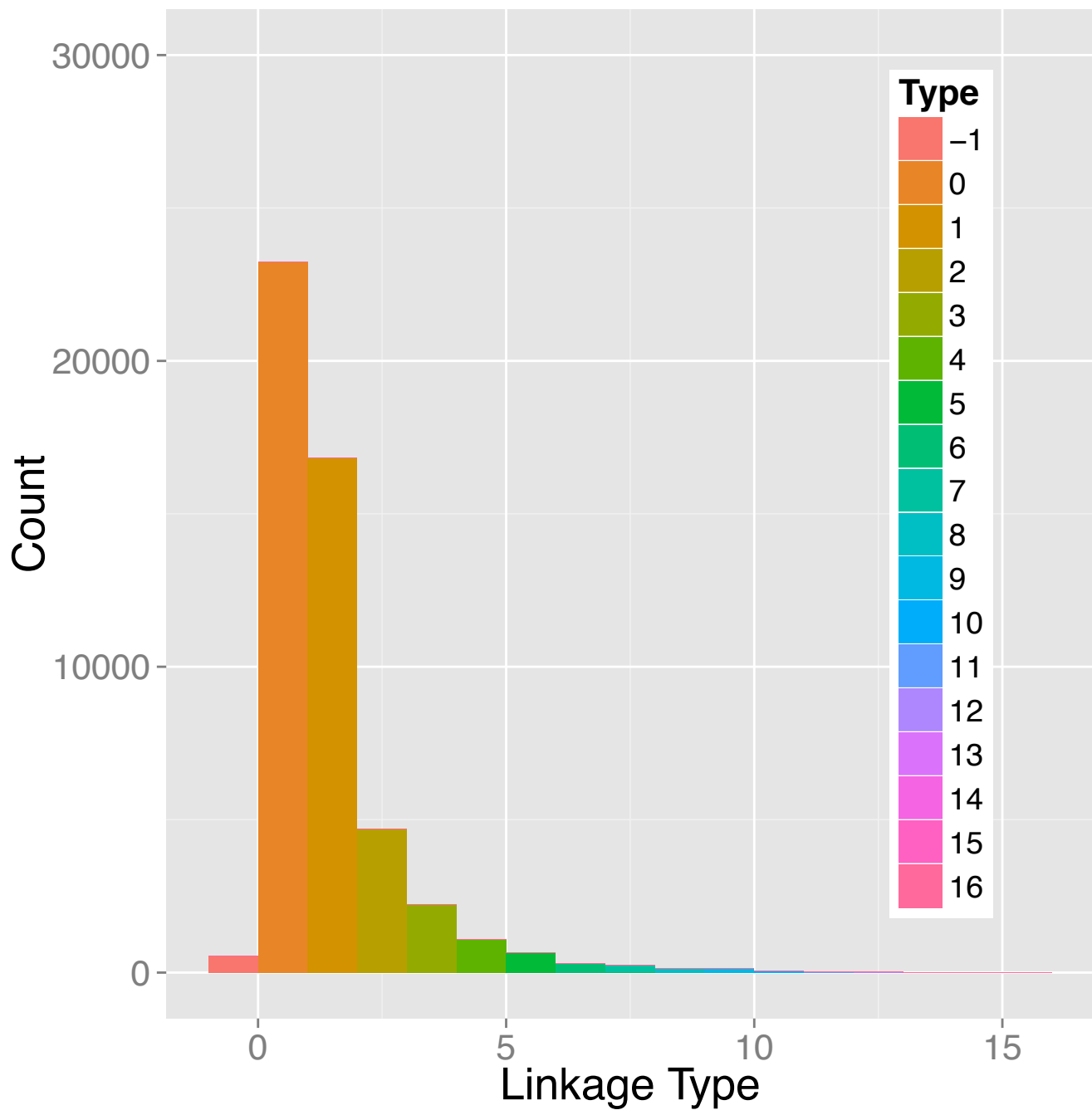

Supplement: Additional file 2: Figure S2. — Distribution of linkage types for chromosome 1. The frequency of linkage types were: 46.3 % unlinked (type 0), 33.5 % linked to a single SNP (type 1), 19.1 % linked to a block where the block showed a majority effect sign (type ≥ 2, where the type number refers to the number of SNPs in the block), and 1.1 % where the block had no majority effect sign (type −1). (PDF 24 kb) [file 12864_2015_1874_MOESM2_ESM.pdf]

**A**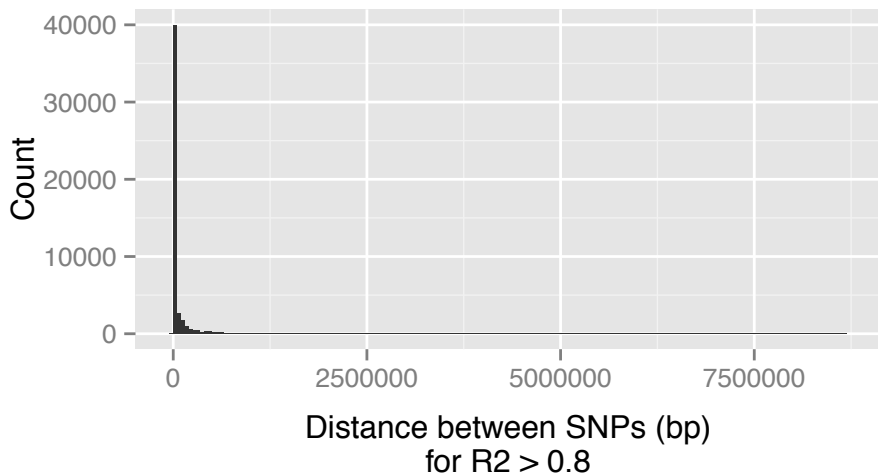**B**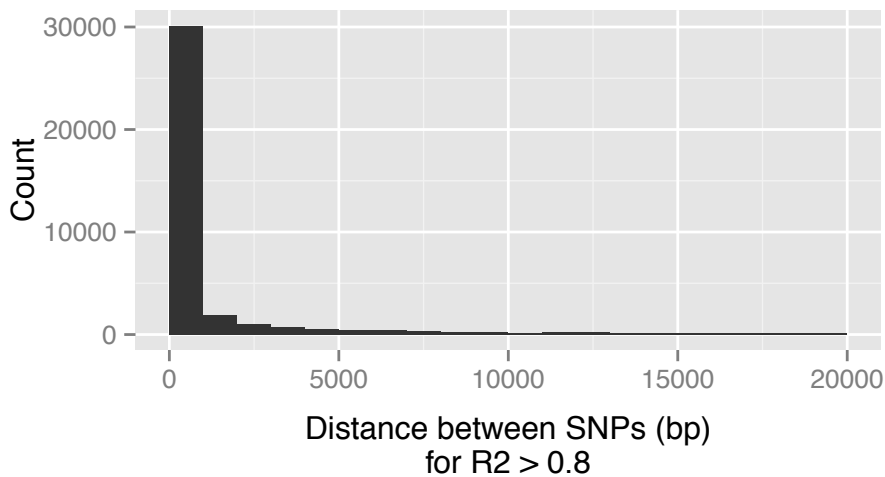

Supplement: Additional file 3: Figure S3. — Histogram of the distance between two linked SNPs for (A) all of chromosome 1, and (B) for the region in (A) where the SNPs were separated by less than 20 Kb. (PDF 41 kb) [file 12864_2015_1874_MOESM3_ESM.pdf]
